# Pediatric Pleural Empyema: Disease Complexity, Contemporary Management, and Future Research Priorities

**DOI:** 10.3390/jcm15218205

**Published:** 2026-10-22

**Authors:** Gabriele Tarditi, Camilla Maria Pisa, Ilaria Ricci, Raffaella Civino, Giorgia Montis, Valentina Fainardi, Susanna Esposito

**Affiliations:** Pediatric Clinic, Department of Medicine and Surgery, University Hospital of Parma, 43126 Parma, Italy; gabriele.tarditi@unipr.it (G.T.); camillamaria.pisa@unipr.it (C.M.P.); ilaria.ricci@unipr.it (I.R.); raffaella.civino@unipr.it (R.C.); giorgia.montis@unipr.it (G.M.); valentina.fainardi@unipr.it (V.F.)

**Keywords:** pediatric pleural empyema, complicated parapneumonic effusion, intrapleural fibrinolysis, pleural drainage, video-assisted thoracoscopic surgery, antimicrobial therapy

## Abstract

**Background**: Pediatric pleural empyema remains a major complication of community-acquired pneumonia despite vaccination and advances in minimally invasive treatment. This narrative review summarizes current evidence on etiology and microbiology, diagnosis and imaging, antimicrobial therapy, supportive care, pleural drainage, fibrinolysis, surgery, complications, and short- and long-term outcomes. **Methods**: This structured narrative review searched PubMed from inception through 30 July 2026. No formal risk-of-bias assessment was performed. Of 42 publications assessed in full, 38 were included in the narrative synthesis. **Results**: *Streptococcus pneumoniae*, particularly serotype 3, *Staphylococcus aureus*, and *Streptococcus pyogenes* remain the principal pathogens. Chest ultrasound is preferred for characterizing pleural collections and guiding intervention, whereas computed tomography is reserved for atypical disease or suspected complications. Small pediatric comparative trials report broadly similar outcomes for catheter drainage with fibrinolysis and video-assisted thoracoscopic surgery (VATS), but differences in sample size, eligibility, treatment protocols, and outcome definitions limit conclusions about comparative effectiveness. Image-guided small-bore catheter drainage with protocol-based urokinase or tissue plasminogen activator is effective for many loculated effusions. Pediatric evidence does not support routine addition of DNase. Comparative trials show broadly similar clinical outcomes for fibrinolysis and video-assisted thoracoscopic surgery, although heterogeneous protocols and limited sample sizes preclude identification of a universally superior strategy. Thoracotomy is rarely required. **Conclusions**: Management should integrate respiratory and septic severity, pleural anatomy, microbiology, adequacy of drainage, clinical trajectory, and local expertise rather than rely on rigid staging. Long-term recovery is excellent in most previously healthy children receiving timely treatment. Standardized definitions, pediatric outcome sets, multicenter comparative trials, validated predictors of treatment failure, and studies of antimicrobial duration remain important research priorities.

## 1. Introduction

Pediatric pleural infection encompasses a heterogeneous clinical spectrum ranging from uncomplicated parapneumonic effusion to complicated effusion and frank empyema. Although pleural empyema is conventionally defined by the presence of purulent pleural fluid or microbiological evidence of infection, many children present with culture-negative, loculated collections that are clinically managed within the same disease continuum. Inconsistent terminology, variable diagnostic criteria, and differing definitions of disease severity and treatment failure continue to limit comparisons among studies and contribute to substantial variation in reported incidence, therapeutic thresholds, and outcomes [1,2,3,4,5].

The conventional division of pleural infection into exudative, fibrinopurulent, and organizing phases provides a useful pathophysiological framework but only partially reflects the complexity encountered in clinical practice. These phases may overlap, and neither imaging appearances nor symptom duration reliably define the biological stage or reversibility of the disease. Consequently, the management of pediatric empyema cannot be reduced to a rigid stage-based algorithm and instead requires integration of clinical severity, pleural anatomy, systemic inflammatory response, associated pulmonary disease, and evolution over time [2,3,5,6]. This complexity also helps explain why apparently similar patients may receive markedly different treatments across institutions.

A further reason for reassessing the available evidence is the rapidly changing epidemiological and microbiological context. Pneumococcal conjugate vaccination has modified the incidence and serotype distribution of pediatric pleural infection without eliminating its burden. At the same time, persistent pneumococcal disease, the continuing importance of *Staphylococcus aureus*, and recent changes in invasive *Streptococcus pyogenes* infections have altered the considerations underlying empirical antimicrobial treatment [7,8,9,10,11,12]. Conventional culture methods frequently fail to identify the causative organism, particularly after antibiotics have been administered, while molecular techniques have improved pathogen detection and challenged some conclusions derived from culture-based studies [4,11,13]. Evidence generated in different geographical areas or vaccine eras may therefore not be directly transferable to contemporary practice.

Despite the publication of guidelines, observational studies, randomized trials, and previous reviews, several reasons justify an updated critical synthesis. The available literature remains fragmented across infectious-disease, respiratory, radiological, interventional, and surgical disciplines. Comparative studies are generally small, use heterogeneous eligibility criteria and treatment protocols, and assess outcomes that are not consistently defined [1,5,14,15,16,17,18,19,20]. Moreover, some therapeutic practices have been influenced by adult studies, although differences in disease biology, comorbidity, prognosis, and treatment response limit direct extrapolation to children [21,22,23]. As a result, areas of genuine uncertainty may be obscured by recommendations that appear more definitive than the underlying pediatric evidence permits.

A contemporary review should do more than describe the available diagnostic and therapeutic options. It should clarify the quality and applicability of the supporting evidence, reconcile differences among studies and recommendations, and distinguish established principles from practices based mainly on observational data, institutional preference, or extrapolation from adults. This narrative review brings together evidence from infectious-disease, respiratory, radiological, interventional, and surgical perspectives. It emphasizes how clinical severity, pleural anatomy, microbiology, complications, and local expertise shape management decisions, and identifies areas where pediatric evidence remains insufficient to guide practice.

## 2. Methods

### 2.1. Review Design and Scope

This study was conducted as a structured narrative review of the contemporary literature concerning pediatric pleural empyema. The review was designed to provide a clinically oriented synthesis of disease epidemiology, microbiology, diagnosis, antimicrobial treatment, supportive care, pleural drainage, intrapleural fibrinolysis, surgical management, complications, and outcomes. Because the objective was to integrate evidence from heterogeneous study designs and address areas in which pediatric comparative data remain limited, the review was not designed as a systematic review or meta-analysis. No formal review protocol was registered.

### 2.2. Information Source and Search Strategy

PubMed was searched from database inception through July 2026, without restrictions on publication date. The search combined terms describing pleural infection with terms related to the pediatric population and relevant therapeutic interventions. Search terms included “pleural empyema,” “empyema,” “pleural infection,” “parapneumonic effusion,” “complicated parapneumonic effusion,” “pediatric,” “paediatric,” “child,” “children,” “infant,” “adolescent,” “antibiotic therapy,” “antimicrobial therapy,” “antibiosis,” “pleural drainage,” “chest tube,” “thoracostomy,” “fibrinolysis,” “urokinase,” “alteplase,” “tissue plasminogen activator,” “DNase,” “video-assisted thoracoscopic surgery,” “VATS,” “thoracoscopy,” “thoracotomy,” “decortication,” “necrotizing pneumonia,” and “bronchopleural fistula.” Boolean operators were used to combine disease-, population-, and intervention-related concepts. The search was limited to studies involving patients aged 0–18 years whenever an appropriate database filter was available.

To identify publications not retrieved through the electronic search, the reference lists of included articles, relevant reviews, clinical practice guidelines, and consensus documents were examined manually. Recent publications were also reviewed to identify studies that updated or extended findings from earlier pediatric cohorts. The final PubMed search was conducted on 30 July 2026. Forty-two full-text publications were assessed for eligibility. Four were excluded because they were duplicate publications or did not provide information relevant to the objectives of the review. The remaining 38 publications were included in the narrative synthesis.

### 2.3. Eligibility Criteria

Peer-reviewed English-language publications were considered eligible when they addressed at least one clinically relevant aspect of pediatric pleural empyema or complicated parapneumonic effusion. Eligible topics included epidemiology; vaccine-era changes in pathogen distribution; conventional and molecular microbiological diagnosis; radiographic, ultrasonographic, computed-tomographic, or magnetic-resonance imaging; empirical and targeted antimicrobial therapy; supportive care; thoracentesis; chest-tube drainage; intrapleural fibrinolysis; video-assisted thoracoscopic surgery (VATS); thoracotomy or decortication; necrotizing pneumonia; bronchopleural fistula; treatment-related adverse events; healthcare utilization; and short- or long-term clinical outcomes.

Randomized controlled trials, non-randomized comparative studies, prospective and retrospective observational studies, surveillance studies, cohort studies, clinically relevant case series, systematic reviews, narrative reviews, clinical practice guidelines, and consensus statements were considered. Priority was given to original pediatric studies, particularly randomized or comparative investigations, multicenter cohorts, national surveillance studies, and recent guidelines developed using formal evidence-assessment methods.

Publications were excluded if they focused exclusively on adults without providing information necessary to interpret pediatric practice; did not report findings relevant to pleural infection or its management; consisted only of conference abstracts, editorials, correspondence, or expert opinion without supporting data; duplicated previously published populations without contributing additional relevant information; or lacked sufficient methodological or clinical information for interpretation. Studies combining adult and pediatric participants were considered only when pediatric data could be identified separately.

Because some therapies used in children were originally evaluated in adults, selected foundational adult studies were retained when they directly informed a major area of pediatric practice or highlighted the limitations of extrapolating adult evidence to children. These studies were clearly distinguished from pediatric evidence in the narrative synthesis and were not used to establish pediatric treatment superiority in the absence of supporting pediatric data. This approach was particularly relevant to combined intrapleural tissue plasminogen activator and DNase therapy.

### 2.4. Study Selection

Search results were imported into Rayyan (Rayyan Systems Inc., Cambridge, MA, USA; https://rayyan.ai, accessed on 11 October 2026) for record management and eligibility assessment. Duplicate records were removed before screening. Titles and abstracts were initially evaluated for relevance, after which potentially eligible publications underwent full-text assessment. Eligibility was determined according to the predefined population, topic, study-design, and publication-type criteria. Two reviewers (G.T. and C.M.P.) independently screened titles and abstracts and assessed potentially eligible full-text publications against the predefined criteria. Disagreements were resolved through discussion and consensus; a third reviewer (I.R.) was consulted when consensus could not be reached.

A total of 42 publications underwent full-text assessment. Four were excluded because they were duplicate publications or did not provide information relevant to the objectives of the review. The remaining 38 publications were included in the final narrative synthesis. Because this was a narrative review, a formal PRISMA flow diagram was not considered mandatory; nevertheless, the numbers of full-text publications assessed, excluded, and included are reported to improve transparency.

### 2.5. Data Extraction and Evidence Organization

For each included publication, relevant information was collected on study design, country or geographical setting, study period, patient age and selection criteria, number of participants, definition of parapneumonic effusion or empyema, microbiological methods, imaging approach, treatment strategy, comparator, outcome definitions, follow-up duration, and principal findings. For interventional studies, additional attention was given to chest-tube characteristics, fibrinolytic agent and regimen, timing and type of surgery, crossover between treatment groups, adverse events, and criteria used to define treatment failure or escalation.

The extracted evidence was organized into the following domains: etiology and microbiology; diagnosis and imaging; antimicrobial therapy and supportive care; pleural drainage and intrapleural fibrinolysis; surgical treatment; necrotizing pneumonia and bronchopleural fistula; and short- and long-term outcomes. Evidence was summarized descriptively because substantial clinical and methodological heterogeneity precluded meaningful quantitative pooling.

### 2.6. Assessment and Interpretation of the Evidence

No formal risk-of-bias instrument or certainty-of-evidence framework was applied, consistent with the narrative design of the review. Nevertheless, evidence was interpreted according to study design, sample size, patient-selection methods, completeness of follow-up, appropriateness of comparator groups, consistency of outcome definitions, and relevance to contemporary pediatric practice. Recent pediatric guidelines were used to organize current recommendations on ultrasound, pleural drainage, catheter size, fibrinolysis, and drainage versus surgical debridement [1,6,14,21]. Pediatric randomized trials were prioritized for direct comparisons of fibrinolytic therapy and VATS [17,18,19,20], whereas contemporary national or multicenter surveillance studies informed pathogen distribution and antimicrobial practice [7]. Adult evidence was used to explain the origin of selected practices, such as combined tissue plasminogen activator (tPA, alteplase)–DNase treatment, but was not treated as proof of benefit in children when pediatric trial data were available [22,23].

Particular attention was paid to sources of heterogeneity that could explain conflicting results. These included inconsistent definitions of complicated parapneumonic effusion and empyema, differences in disease severity and duration before intervention, variable imaging criteria, non-standardized fibrinolytic regimens, differences in surgical expertise, treatment crossover, institutional escalation thresholds, and variation in definitions of clinical success or treatment failure. Findings derived exclusively from adults were not assumed to be directly applicable to children.

### 2.7. Narrative Synthesis

The evidence was synthesized using a clinically oriented framework centered on the principal decisions encountered during management: selection and de-escalation of antimicrobial therapy, identification of children requiring pleural drainage, choice of drain and fibrinolytic regimen, recognition of inadequate source control, selection and timing of VATS, and indications for thoracotomy or formal decortication. Areas supported by pediatric randomized or comparative evidence were distinguished from those based predominantly on observational studies, expert guidance, or extrapolation from adult pleural infection. Remaining uncertainties were used to define priorities for future pediatric research.

## 3. Etiology and Microbiology

Pediatric pleural empyema most commonly develops as a complication of bacterial pneumonia. Inflammation of the adjacent lung increases pleural vascular permeability and produces an initially sterile parapneumonic effusion. Bacterial invasion of the pleural space subsequently amplifies the inflammatory response, with neutrophil recruitment, activation of coagulation, inhibition of endogenous fibrinolysis, and fibrin deposition. The resulting septations and loculations impair pleural-fluid clearance and may lead to the accumulation of purulent material. Persistent inflammation promotes fibroblast proliferation and collagen deposition, potentially producing an organizing pleural peel that restricts lung expansion [2,3,24]. The rate and extent of progression depend on pathogen virulence, bacterial burden, host inflammatory response, duration of illness, and timing of effective antimicrobial treatment.

Pneumonia accounts for the great majority of pediatric cases, but pleural infection may occasionally arise through contiguous spread, hematogenous dissemination, trauma, or iatrogenic contamination. Potential sources include pulmonary abscess, mediastinal or subdiaphragmatic infection, vertebral or rib osteomyelitis, esophageal perforation, thoracic injury, surgery, and pleural instrumentation. Secondary infection of a pre-existing effusion may also occur in children with malignancy, severe systemic disease, or postoperative pleural collections [3,6,25]. These alternative mechanisms should be considered when the presentation is atypical, infection is polymicrobial or anaerobic, or the response to conventional treatment is unsatisfactory.

The microbiology of pediatric parapneumonic effusion and empyema is dominated by Gram-positive aerobic cocci (Table 1) [1,3,4,6,7,8,9,10,11,12,13,14,25,26,27].

*Streptococcus pneumoniae* remains the leading pathogen in many settings despite widespread pneumococcal conjugate vaccination [8,25]. Introduction of PCV7 and subsequently PCV13 reduced disease caused by covered serotypes and was associated with declining empyema hospitalization rates; however, the magnitude and durability of this effect have varied geographically [7,8,9,10]. Serotype replacement and incomplete protection against individual serotypes have maintained a residual burden of pneumococcal pleural disease. Serotype 3 is of particular concern because it continues to cause complicated pneumonia and empyema in fully vaccinated children, possibly because of its abundant polysaccharide capsule and capacity to induce an intense inflammatory response [9,10]. However, pneumococcal serotype distribution and the observed effects of pneumococcal conjugate vaccines are population- and time-specific. Reports of persistent serotype 3 disease and serotype replacement should therefore be attributed to the surveillance populations and periods from which they were derived, rather than presented as uniform global patterns. Vaccine uptake, vaccine formulation, local epidemiology, and surveillance methods differ among countries. The effect of newer higher-valency vaccines on pediatric empyema incidence and serotype distribution remains to be established through continued, geographically diverse surveillance.

*Staphylococcus aureus* and *Streptococcus pyogenes* are the other principal causes of pediatric empyema. Both methicillin-susceptible and methicillin-resistant S. aureus may cause severe pleuropulmonary infection. Community-associated MRSA should be considered in rapidly progressive pneumonia associated with pulmonary necrosis, pneumatoceles, hemoptysis, or bronchopleural fistula, although its prevalence differs substantially among regions [11]. *Streptococcus pyogenes* may produce rapidly progressive invasive disease accompanied by bacteremia, systemic toxicity, or shock. The recent increase in invasive group A streptococcal infections in several European countries has renewed concern regarding its contribution to complicated pneumonia and empyema [11,12].

Less frequently identified organisms include *Haemophilus influenzae*, members of the *Streptococcus anginosus* group, anaerobic bacteria, and Gram-negative bacilli such as *Klebsiella pneumoniae*, *Pseudomonas aeruginosa*, and *Stenotrophomonas maltophilia* [4,13]. These pathogens are more likely in neonates, immunocompromised children, patients with neurological impairment or aspiration risk, children with chronic comorbidities, and those with healthcare-associated infection or previous broad-spectrum antibiotic exposure. Recovery of anaerobic or multiple organisms should prompt consideration of aspiration, poor dentition, esophageal perforation, trauma, surgery, or extension from a subdiaphragmatic source [3,4].

Identification of the causative organism remains challenging. Blood cultures have limited sensitivity, and pleural-fluid cultures are frequently negative because antibiotics are commonly administered before drainage. Molecular techniques, including species-specific polymerase chain reaction and broad-range bacterial amplification, substantially increase diagnostic yield and have confirmed the continuing importance of pneumococcus in culture-negative empyema [4,11,13]. However, molecular detection does not establish antimicrobial susceptibility and may detect non-viable organisms. Whenever pleural sampling is clinically indicated, fluid should therefore be submitted for Gram staining, aerobic and anaerobic cultures, susceptibility testing, and complementary molecular analysis where available [4,11,26].

Overall, pediatric empyema reflects an interaction among pathogen virulence, host susceptibility, vaccine-induced selective pressure, previous antibiotic exposure, and regional epidemiology. Although *Streptococcus pneumoniae*, *Staphylococcus aureus*, and *Streptococcus pyogenes* account for most cases, the broader microbiological spectrum requires empirical treatment to be individualized according to age, immunization status, disease severity, comorbidities, local resistance patterns, and risk factors for MRSA, anaerobic, Gram-negative, or healthcare-associated infection [1,11,25,26].

## 4. Initial Assessment and Imaging

Initial assessment should establish the child’s respiratory and hemodynamic status, identify evidence of sepsis, and determine whether pleural fluid is likely to be contributing to respiratory compromise. Chest radiography can identify an effusion and provide an initial estimate of its size. For a moderate or large effusion requiring further characterization, ultrasound is preferred to CT or MRI because it can define the size and complexity of the collection, be repeated at the bedside, and guide pleural procedures. Additional imaging is generally unnecessary for a small effusion in a child with minimal respiratory symptoms. CT may be considered when ultrasound does not resolve a clinically important question, or when parenchymal complications or an atypical source are suspected [14].

Pleural-fluid sampling is appropriate when drainage is clinically indicated and can provide material for Gram stain, culture, susceptibility testing, and molecular testing where available. Imaging findings should inform decisions about drainage and procedural access, but should be interpreted together with respiratory status, systemic illness, and clinical course. A septated or loculated collection alone does not establish the need for a particular drainage method [1,5,6,14].

## 5. Therapeutic Approaches

The management of pediatric pleural empyema requires the simultaneous treatment of pulmonary and pleural infection, maintenance of respiratory and hemodynamic stability, and effective pleural source control when a clinically significant infected collection is present. Treatment should be individualized according to respiratory status, effusion size and organization, duration of illness, microbiological findings, associated necrotizing pneumonia or bronchopleural fistula, response to initial therapy, and local interventional-radiological and surgical expertise. The pathophysiological continuum and the principal clinical factors that should guide management are summarized in Figure 1.

The traditional three-stage classification provides a useful pathophysiological framework but should not be used as the sole determinant of treatment because clinical, ultrasonographic, operative, and histopathological stages may not correspond precisely [1,2,5,6].

Figure 2 summarizes the principal components of the stepwise multidisciplinary approach, from initial stabilization and empirical antimicrobial treatment to pleural drainage, intrapleural fibrinolysis, and surgical escalation.

The 2026 IDSA/PIDS recommendations [1,6,14,21] address community-acquired pneumonia with parapneumonic effusion in children aged 3 months to 18 years. They should not be assumed to apply directly to neonates or to all healthcare-associated, postoperative, aspiration-related, or other non-pneumonic pleural infections. Neonatal empyema requires separate age-specific assessment of likely pathogens, antimicrobial coverage, and source control.

### 5.1. Supportive Care

Supportive care should be initiated concurrently with antimicrobial therapy and assessment of the need for pleural drainage. Continuous clinical evaluation is essential because respiratory impairment may result from a combination of pleural compression, parenchymal consolidation, necrotizing pneumonia, pain-related splinting, sepsis, or bronchopleural air leak. Supplemental oxygen is indicated for hypoxemia, while high-flow nasal oxygen, non-invasive ventilation, or invasive mechanical ventilation may be required in severe disease. Decisions regarding drainage or escalation of respiratory support should therefore be based on the overall clinical trajectory rather than effusion size alone [1,2,6].

Fluid management should balance correction of dehydration against the risk of fluid overload. Isotonic intravenous fluids should be adjusted according to perfusion, urine output, electrolyte concentrations, oral intake, and ongoing losses [1]. Particular attention should be paid to hyponatremia, which may result from inflammation-associated inappropriate antidiuretic hormone secretion. Nutritional assessment and early nutritional support should be considered when fever, respiratory distress, pain, or prolonged hospitalization substantially limits oral intake [1].

Pain is a major component of pleural infection and may be intensified by chest-tube placement, drain manipulation, intrapleural fibrinolysis, and surgery. Inadequate analgesia promotes shallow breathing, ineffective coughing, immobility, and poor tolerance of pleural procedures [27]. A multimodal strategy using paracetamol and, when not contraindicated, non-steroidal anti-inflammatory drugs is appropriate for most children; opioids or regional anesthetic techniques may be required for severe pain or postoperative care. Analgesia should be administered proactively before drain manipulation or fibrinolytic instillation. Mobilization, position changes, and age-appropriate breathing exercises may support recovery once pain is controlled, whereas routine aggressive chest physiotherapy during the acute phase has not been shown to accelerate pleural-fluid resolution and may be poorly tolerated [5].

Children should be reassessed regularly for persistent fever, increasing oxygen requirement, hemodynamic instability, reduced drain output, new air leak, or worsening inflammatory markers [27,28]. These findings should not automatically be attributed to inadequate antimicrobial therapy; they may indicate catheter obstruction or malposition, an undrained loculation, progressive parenchymal necrosis, pulmonary abscess, bronchopleural fistula, or another infectious focus. Early multidisciplinary review involving pediatrics, infectious diseases, respiratory medicine, interventional radiology, and pediatric surgery may prevent delays in achieving adequate source control.

The role of systemic corticosteroids remains uncertain. Limited pediatric evidence, including a randomized study of dexamethasone, suggests possible reductions in inflammatory markers and hospital length of stay [29,30]. However, the available studies are insufficient to support routine corticosteroid treatment because of restricted sample sizes, heterogeneous patient selection, and variation in concomitant therapies and outcome definitions. Potential benefits must be weighed against adverse effects and the possibility of obscuring persistent infection. Corticosteroids should therefore be considered an investigational or selectively applied adjunct rather than a standard component of empyema management.

### 5.2. Antibiotic Therapy

Antimicrobial treatment should be initiated promptly when complicated parapneumonic effusion or empyema is suspected and should not be delayed while awaiting drainage or microbiological confirmation. Blood cultures should be obtained before the first antibiotic dose whenever this does not postpone treatment. When pleural intervention is clinically indicated, fluid should be submitted for Gram staining, aerobic and anaerobic cultures, antimicrobial susceptibility testing, and molecular analysis where available. Because antibiotics are frequently administered before pleural sampling, conventional cultures have limited sensitivity; molecular testing can substantially improve pathogen detection, particularly in culture-negative pneumococcal disease [1,4,11,26].

Empirical treatment should provide activity against *Streptococcus pneumoniae*, *Staphylococcus aureus*, and *Streptococcus pyogenes*. In a fully immunized, previously healthy child with community-acquired infection and no risk factors for resistant organisms, appropriate initial regimens generally include a second- or third-generation cephalosporin, such as cefuroxime, cefotaxime, or ceftriaxone, or an aminopenicillin combined with a beta-lactamase inhibitor, such as ampicillin–sulbactam or amoxicillin–clavulanate [1,5,26,27]. Selection should reflect local pneumococcal susceptibility, vaccination coverage, recent antibiotic exposure, institutional protocols, and the severity of presentation.

Additional anti-staphylococcal coverage should be considered in children with septic shock, rapidly progressive pneumonia, influenza-associated disease, pulmonary necrosis, pneumatoceles, hemoptysis, bronchopleural fistula, Gram-positive cocci in clusters, or epidemiological risk factors for MRSA. Vancomycin is commonly used when MRSA is strongly suspected. Clindamycin may be an alternative in clinically stable children when local resistance is low and susceptibility—including the absence of inducible resistance—has been established. Once methicillin-susceptible *Staphylococcus aureus* is identified, treatment should be narrowed to an anti-staphylococcal beta-lactam such as oxacillin, nafcillin, or cefazolin. Vancomycin is not routinely required for penicillin-non-susceptible pneumococcal infection; definitive therapy should instead be guided by the isolate’s susceptibility profile and the child’s clinical response [1,26,27].

Routine addition of a macrolide, aminoglycoside, or clindamycin to an otherwise appropriate beta-lactam regimen has not demonstrated improved outcomes in unselected pediatric empyema. Surveillance data indicate substantial use of empirical combination therapy without evidence of superior clinical outcomes, although confounding by indication and disease severity limits causal interpretation [27]. Combination therapy should therefore be reserved for a defined indication, such as suspected MRSA, toxin-mediated invasive streptococcal or staphylococcal disease, documented atypical coinfection, or polymicrobial infection. Broader Gram-negative or anaerobic coverage may be necessary in neonates, immunocompromised children, aspiration-related disease, hospital-acquired infection, esophageal perforation, thoracic trauma, postoperative empyema, or infection extending from an intra-abdominal source [3].

Antimicrobial therapy should be narrowed once culture, molecular, and susceptibility results become available. In culture-negative disease, de-escalation should consider the most likely pathogen, local epidemiology, initial disease severity, adequacy of pleural source control, and clinical response. Persistent fever during the first days of effective treatment is common and does not alone demonstrate antibiotic failure. Conversely, persistent or recurrent fever accompanied by respiratory deterioration, rising or non-improving inflammatory markers, limited drain output, or a residual pleural collection should prompt reassessment for inadequate source control, catheter dysfunction, resistant or unexpected organisms, necrotizing pneumonia, lung abscess, bronchopleural fistula, or another infectious focus [1,2,5].

The optimal duration of antimicrobial treatment has not been established in randomized pediatric trials. Total courses of approximately 2–4 weeks are commonly used, although treatment should be individualized according to pathogen, disease severity, source-control adequacy, complications, and clinical response [1,5,25]. Prolonged intravenous treatment is not required solely because an empyema was present. Transition to an active oral agent may be considered when the child is hemodynamically stable, respiratory support is no longer required or is clearly decreasing, fever and systemic symptoms are resolving, oral intake is adequate, inflammatory markers show a consistent downward trend, and pleural drainage is satisfactory. Radiographic abnormalities often persist after clinical recovery and should not, in isolation, determine antibiotic duration.

Antimicrobial stewardship is particularly important because broad-spectrum and multidrug regimens are frequently continued despite negative cultures and clinical improvement. Daily reassessment of spectrum, route, and duration can reduce unnecessary exposure without compromising outcomes. Table 2 summarizes the principal microbiological contexts and their implications for empirical and definitive antimicrobial therapy.

### 5.3. Pleural Drainage and Intrapleural Fibrinolytic Therapy

The decision to drain a parapneumonic effusion should be based on respiratory status, effusion size and organization, evidence of pleural infection, and response to antimicrobial therapy. Small, free-flowing effusions in clinically stable children can usually be managed with antibiotics and close observation. Drainage is generally indicated for respiratory compromise, a large pleural collection, macroscopically purulent fluid, microbiological evidence of pleural infection, or persistent systemic illness despite appropriate antimicrobial therapy [1,5,6]. Loculation supports the need for active source control when accompanied by ongoing clinical illness, but septations on ultrasound alone do not determine the treatment modality. Pleural-fluid pH and glucose thresholds derived from adult practice are less well validated in children and should not replace integrated clinical and ultrasonographic assessment.

Repeated therapeutic thoracentesis is seldom appropriate for an established infected collection because it does not provide continuous drainage and may expose the child to multiple procedures and sedations. Ultrasound-guided catheter placement allows targeted drainage and microbiological sampling while avoiding ionizing radiation. Small-bore catheters, including pigtail drains, are generally preferred because they are less painful and appear to achieve outcomes comparable to those of larger tubes when correctly positioned and maintained [5,31]. Drainage effectiveness depends primarily on catheter position and patency and on whether all clinically relevant loculations communicate with the drain.

Drain function should be evaluated through serial assessment of respiratory status, fever, inflammatory markers, drain output, air leak, and residual pleural fluid. Ultrasound is useful when persistent or recurrent fluid is suspected, whereas routine daily chest radiography is unnecessary in a clinically improving child [5,14]. An abrupt reduction in output should prompt assessment for catheter obstruction, kinking, malposition, or isolation of the drain from residual loculations. The use of suction, drain flushing, and temporary clamping varies among institutions because high-quality pediatric comparative evidence is limited. Such practices should follow standardized local protocols and be adapted to drainage dynamics and patient tolerance rather than rigid volume-based rules.

Intrapleural fibrinolysis is used to improve drainage when fibrinous septations and loculations prevent effective evacuation through the catheter. Urokinase and tissue plasminogen activator (tPA; alteplase) are the agents most frequently used in children [5,21,32,33]. Pediatric trials indicate that catheter drainage combined with fibrinolysis achieves clinical outcomes broadly comparable to those of primary video-assisted thoracoscopic surgery (VATS), while avoiding an initial thoracic operation in many patients [16,17,18,19,20]. However, available studies are relatively small and use heterogeneous definitions, dosing protocols, and escalation criteria.

Urokinase has the longest randomized-trial experience in children. A commonly cited regimen consists of twice-daily administration for 3 days, using 10,000 units in 10 mL of 0.9% saline in children weighing less than 10 kg and 40,000 units in 40 mL in those weighing at least 10 kg [5,18]. Alteplase regimens are more variable and generally use weight-based dosing with a defined maximum dose. No agent, dose, administration frequency, or dwell time has been conclusively shown to be superior in pediatric empyema [21,32,33]. Treatment should therefore follow an established pediatric protocol; adult dosing regimens should not be transferred directly to children.

The benefit of adding DNase to fibrinolytic therapy differs between adults and children. Although combined tPA and DNase improved pleural drainage in an adult trial [23], a pediatric randomized trial found that addition of DNase to tPA did not reduce hospital length of stay, treatment failure, or other relevant outcomes compared with tPA alone [21,22]. Routine tPA–DNase combination treatment is therefore not supported by current pediatric evidence.

Pain, transient fever, and blood-stained pleural drainage are the most frequently reported adverse effects of fibrinolysis. Major pleural bleeding and hemothorax are uncommon but potentially serious. Active bleeding, clinically significant uncorrected coagulopathy, or a substantial bleeding risk requires individualized assessment before treatment. Bronchopleural fistula or a major air leak also warrants caution because intrapleural medication may communicate with the bronchial tree. Necrotizing pneumonia alone is not necessarily an absolute contraindication, but associated cavitation, hemoptysis, friable lung, or bronchopleural fistula should prompt multidisciplinary evaluation [2,5,21,32].

Reassess immediately if respiratory support needs rise, hypoxemia or respiratory distress worsens, hemodynamic instability develops, a new or increasing air leak appears, or the child shows other signs of clinical deterioration. In a stable child, reassess drain position and patency, output, fever, inflammatory markers, respiratory status, and residual fluid within approximately 24–48 h. Persistent fever alone during this interval does not establish treatment failure. If the child is clinically stable or improving and drainage is adequate, the planned fibrinolytic course can generally be completed with continued monitoring. Worsening illness, an obstructed or malpositioned catheter, or a substantial residual collection with inadequate drainage should prompt earlier evaluation for catheter revision, additional drainage, or surgical escalation [7,34].

### 5.4. Chest Surgery

Surgical management of pediatric empyema has shifted from open thoracotomy toward minimally invasive thoracoscopic treatment. VATS permits direct evacuation of pus, disruption of loculations, removal of loose fibrinous material, pleural irrigation, microbiological sampling, assessment of lung expansion, and placement of chest drains under direct vision [16,35]. Thoracoscopic debridement should be distinguished from formal decortication: removal of a dense visceral pleural peel is required only when organization substantially prevents lung expansion. Unnecessarily aggressive dissection of acutely inflamed or friable pleura may cause bleeding, parenchymal injury, or prolonged air leak.

VATS may be selected as the initial source-control procedure or used after failure of catheter drainage and fibrinolysis. Potential indications for an early operative approach include a complex collection that cannot be safely or effectively accessed percutaneously, extensive organization with restricted lung expansion, associated intrathoracic pathology requiring operative assessment, or severe disease in which rapid definitive source control is considered necessary [7,16,34,35]. More commonly, VATS is performed when an appropriately positioned and functioning drain, with protocol-based fibrinolysis when indicated, fails to achieve clinical improvement. Persistent fever alone is insufficient to define treatment failure; continued sepsis, respiratory compromise, inadequate drainage, or a substantial residual collection provides stronger evidence of ineffective source control.

Loculations on ultrasound do not independently mandate surgery because many septated collections respond to catheter drainage and fibrinolysis. Conversely, prolonged conservative management should not continue when the drain is ineffective and the child remains systemically unwell. Before escalation, clinicians should determine whether failure reflects catheter obstruction or malposition, an undrained loculation, resistant or unexpected infection, necrotizing pneumonia, pulmonary abscess, or bronchopleural fistula. Early multidisciplinary discussion among pediatricians, infectious-disease specialists, interventional radiologists, respiratory physicians, and pediatric surgeons can distinguish technical drainage failure from disease requiring operative treatment.

Randomized pediatric trials comparing primary VATS with catheter drainage plus fibrinolysis have generally reported similar rates of clinical recovery and comparable hospital length of stay [16,17,18,19,20]. Fibrinolysis is less invasive, avoids an initial operation and general anesthesia, and may reduce costs, but a proportion of children subsequently require surgery. VATS provides direct mechanical clearance and may shorten the interval to effective source control in selected patients, but it requires general anesthesia, operating-room resources, and pediatric thoracic surgical expertise. Interpretation of the comparative evidence is limited by small sample sizes, variation in fibrinolytic regimens, treatment crossover, inconsistent definitions of failure, and differences in institutional practice [16,17,18,19,20,32,36]. Current evidence therefore supports therapeutic equipoise rather than universal superiority of either approach.

Four pediatric randomized trials have compared catheter drainage with fibrinolysis and VATS. Their sample sizes, eligibility criteria, interventions, endpoints, and results are summarized in Table 3.

Selection between fibrinolysis and VATS should account for respiratory and septic severity, duration of illness, pleural anatomy, accessibility of the collection, associated necrotizing disease or air leak, anesthetic risk, available expertise, and family preferences. Institutional pathways can reduce delays and unwarranted variation, but a rigid stage-based algorithm is unlikely to capture the heterogeneity of pediatric pleural infection [34,37].

Open thoracotomy with decortication is now rarely required. It is principally reserved for advanced organized empyema with a restrictive pleural peel that cannot be managed thoracoscopically, persistent pleural sepsis after less invasive interventions, a major bronchopleural fistula requiring repair, or anatomical and technical circumstances in which VATS is unsafe or ineffective [17,18,19,20,23,38]. Although thoracotomy provides wide exposure for debridement and peel removal, it is associated with greater surgical trauma, postoperative pain, and potentially longer recovery. Lung resection should be avoided whenever possible, including in necrotizing pneumonia, because apparently non-viable lung tissue may recover with antimicrobial therapy, drainage, and time.

Overall, pleural drainage, fibrinolysis, and surgery should be regarded as complementary components of a source-control continuum rather than competing treatments. Catheter drainage with fibrinolysis provides an effective initial strategy for many children with loculated empyema, while VATS is appropriate as primary treatment in selected cases and as escalation therapy when percutaneous source control is inadequate. Thoracotomy remains a rescue procedure for uncommon advanced or refractory disease. Because no intervention is uniformly superior across all clinical presentations, treatment should be individualized according to disease complexity, clinical evolution, complications, and institutional expertise [1,6,15,16,17,18,19,20,21,22,31,32,34,35,36,38].

## 6. Necrotizing Pneumonia and Bronchopleural Fistula

Necrotizing pneumonia and bronchopleural fistula can complicate pediatric pleural infection and may contribute to persistent fever, respiratory deterioration, or an air leak. Their possibility should be considered when a child fails to improve despite appropriate antimicrobial treatment and apparently adequate pleural drainage. Reassessment should include drain position and patency, residual or non-communicating collections, and evidence of parenchymal necrosis, abscess, or fistula. Ultrasound is useful for pleural reassessment; computed tomography may be appropriate when parenchymal complications are suspected and are not adequately defined by ultrasound. These findings can affect antimicrobial coverage, the safety of intrapleural treatment, and the timing or type of surgery. Management should be decided with the relevant pediatric, respiratory, radiological, infectious-disease, and surgical teams [1,2,5,11].

## 7. Short- and Long-Term Outcomes

Short-term assessment should include clinical recovery, adequacy of source control, respiratory support, duration of drainage and hospitalization, treatment failure or crossover, adverse events, and readmission. These outcomes are difficult to compare across studies because definitions and follow-up periods vary. Most previously healthy children recover well after timely antimicrobial treatment and pleural source control. However, the evidence summarized in this review does not establish a consistent set of long-term outcomes or follow-up measures. Persistent imaging abnormalities should be interpreted in the context of clinical recovery, and future studies should report longer-term respiratory function and quality of life using standardized measures [1,2,5,16,17,18,19,20].

Table 4 summarizes the principal pediatric evidence and practical considerations for key pleural-management decisions.

## 8. Study Limitations

This review has limitations. It was conducted as a structured narrative review, with no registered protocol and no formal risk-of-bias assessment or certainty-of-evidence grading. The search was limited to PubMed and English-language publications, so relevant studies indexed elsewhere or published in other languages may have been missed. The included literature also varied in study design, patient selection, definitions of empyema and treatment failure, interventions, and outcomes. These limitations preclude quantitative pooling and mean that the synthesis should be interpreted as a clinically oriented summary rather than a systematic estimate of comparative treatment effects.

## 9. Recommendations for Future Research

Future pediatric studies should use standardized definitions of complicated parapneumonic effusion, empyema, treatment failure, and successful source control, and should report a common set of short- and long-term outcomes. Prospective multicenter comparative studies should identify which clinical and anatomical presentations benefit most from catheter drainage, particular fibrinolytic regimens, or early VATS. Further priorities include clarifying the management of necrotizing pneumonia and bronchopleural fistula; establishing antimicrobial duration and criteria for intravenous-to-oral transition; evaluating rapid molecular diagnostics in culture-negative disease; validating predictors of treatment failure; and assessing long-term respiratory function, quality of life, and family impact. Continued surveillance is needed to track pathogen and serotype distribution, antimicrobial resistance, and the effects of higher-valency pneumococcal vaccines.

## 10. Conclusions

Pediatric pleural empyema requires individualized management based on clinical severity, pleural anatomy, microbiology, associated complications, and response to treatment. Ultrasound is the preferred modality for characterizing pleural collections and guiding intervention. Clinically significant infected collections require timely source control, commonly beginning with image-guided catheter drainage and protocol-based fibrinolysis. VATS is appropriate for selected children and when catheter-based treatment does not achieve adequate source control; thoracotomy is rarely required. Pediatric evidence does not establish universal superiority of fibrinolysis or VATS, so treatment decisions should account for the child’s presentation and available expertise. Most previously healthy children recover well when effective antimicrobial therapy and source control are provided promptly.

## Figures and Tables

**Figure 1:**
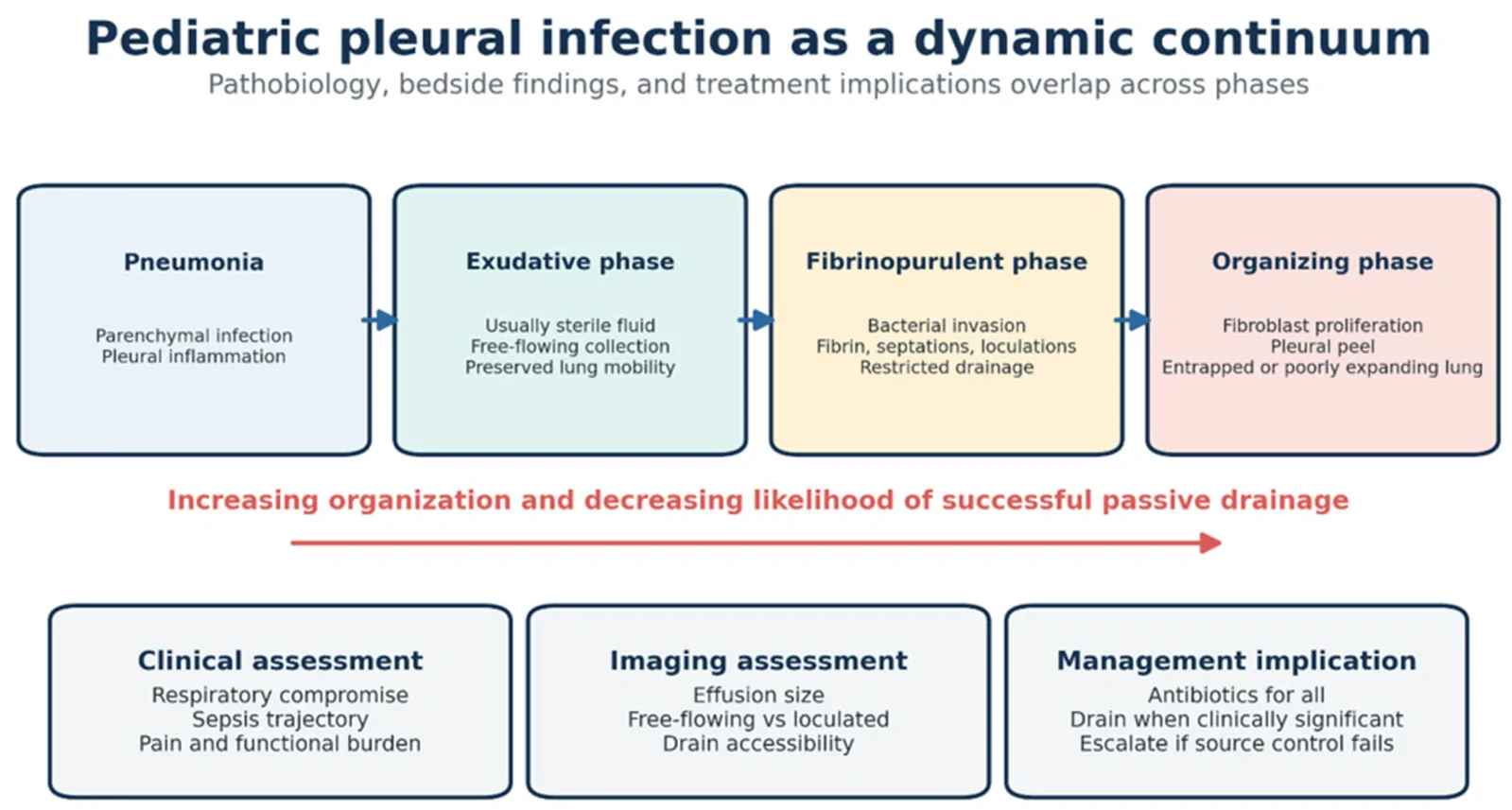
Pediatric pleural infection as a dynamic continuum.

**Figure 2:**
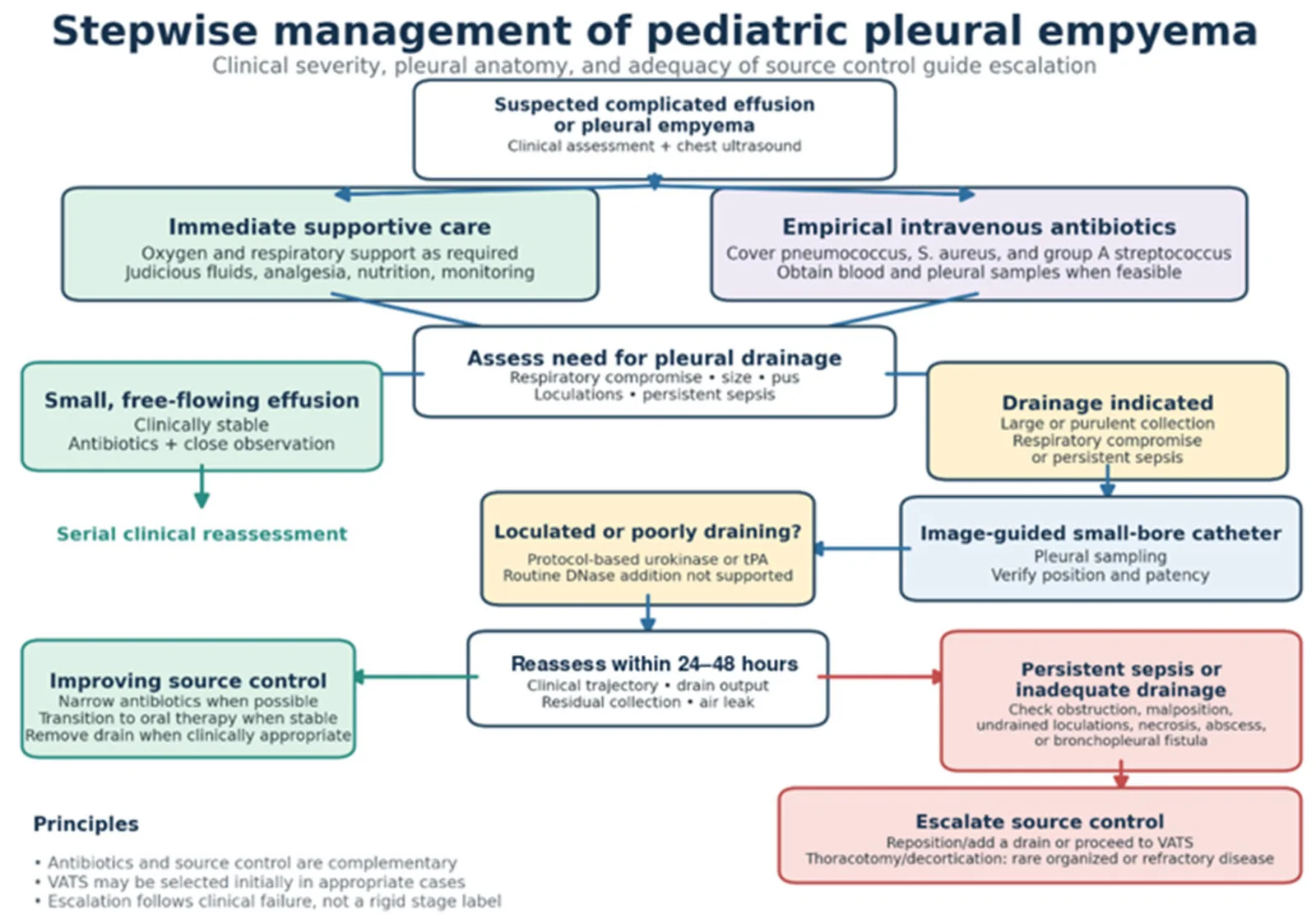
Stepwise management of pediatric pleural empyema.

**Table 1:** Etiological spectrum and clinically relevant features of pediatric pleural empyema.

Organism or Etiological Setting	Epidemiological Context or Risk Factors	Characteristic Clinical Associations	Diagnostic Considerations	Main Clinical Implication
*Streptococcus pneumoniae*	Most frequent cause in many regions; disease persists despite pneumococcal conjugate vaccination	Complicated pneumonia and loculated pleural infection; serotype 3 remains prominent in vaccinated children	Pleural-fluid culture is often negative after antibiotic exposure; molecular testing substantially increases detection	Empirical therapy should retain reliable pneumococcal activity and subsequently be narrowed according to susceptibility findings [4,8,9,10,11,13,25]
*Staphylococcus aureus*, methicillin-susceptible	Community-acquired infection; may occur in previously healthy children	Severe pneumonia, pulmonary necrosis, pneumatoceles, abscess formation, or bronchopleural fistula	Blood and pleural-fluid cultures may be positive; obtain susceptibility testing	Consider early anti-staphylococcal coverage when clinical or radiological features suggest necrotizing disease [1,11]
Methicillin-resistant *S. aureus*	Prevalence varies markedly by geographical region; community- or healthcare-associated exposure	Rapid clinical progression, systemic toxicity, pulmonary destruction, air leak, or hemoptysis	Local resistance data and isolate susceptibility are essential; inducible clindamycin resistance should be assessed when relevant	Empirical MRSA coverage should be guided by disease severity, clinical phenotype, and local epidemiology [1,11,25,26]
*Streptococcus pyogenes*	Increasing invasive disease reported in several European settings; possible association with preceding viral infection	Rapidly progressive pleuropulmonary infection, bacteremia, shock, or marked systemic inflammation	Blood cultures may improve recognition of invasive disease; pleural sampling remains important	Close monitoring and prompt effective therapy are required because clinical deterioration may be rapid [11,12]
*Haemophilus influenzae*	Incomplete vaccination, immunocompromise, or underlying disease; may also occur in vaccinated children	Pneumonia with parapneumonic effusion or empyema	Culture and susceptibility testing are required to assess beta-lactamase production and resistance	Empirical relevance depends on vaccination status and local epidemiology [4]
*Streptococcus anginosus* group	Oral flora, aspiration risk, poor dentition, or contiguous infection	Abscess-forming infection, loculation, and occasionally polymicrobial disease	Anaerobic culture conditions may improve recovery	Consider an oral, aspiration-related, or contiguous infectious source [4,13]
Anaerobic and polymicrobial infection	Aspiration, neurological impairment, poor dentition, esophageal perforation, trauma, surgery, or subdiaphragmatic infection	Foul-smelling or complex pleural infection; atypical or prolonged clinical course	Pleural fluid should be submitted for anaerobic culture before prolonged exposure to oxygen	Broaden investigation beyond uncomplicated community-acquired pneumonia and ensure anaerobic antimicrobial coverage [3,4]
Gram-negative bacilli	Neonatal age, immunocompromise, chronic comorbidity, healthcare exposure, surgery, or previous broad-spectrum antibiotics	Severe or healthcare-associated pleural infection	Culture and susceptibility testing are essential because resistance patterns are variable	Treatment should be individualized according to risk factors, local ecology, and microbiological findings [4,13]
Non-pneumonic empyema	Trauma, thoracic surgery, pleural instrumentation, esophageal perforation, mediastinal or subdiaphragmatic infection, osteomyelitis, malignancy, or pre-existing effusion	Atypical presentation, polymicrobial infection, persistent sepsis, or poor response to standard CAP therapy	Imaging and microbiological investigation should target the underlying anatomical source	Definitive management requires treatment of both the pleural infection and the primary source [3,6,25]

Abbreviations: CAP, community-acquired pneumonia; MRSA, methicillin-resistant *Staphylococcus aureus*.

**Table 2:** Microbiological context and antimicrobial implications.

Pathogen or Setting	Clinical Clues	Initial Coverage	Definitive Approach	Key Considerations
*Streptococcus pneumoniae*	Typical CAP; serotype 3 may cause complicated disease	Ceftriaxone, cefotaxime, or ampicillin–sulbactam	Narrow to a susceptible beta-lactam	Molecular testing improves detection following previous antibiotic exposure [4,7,8,9,10,11,25,26].
Methicillin-susceptible *Staphylococcus aureus*	Severe pneumonia, empyema, pulmonary necrosis, or air leak	Include anti-staphylococcal activity when clinically suspected	Oxacillin, nafcillin, or cefazolin	Pleural source control is essential; evaluate for necrotizing pneumonia or bronchopleural fistula [1,11].
Methicillin-resistant *Staphylococcus aureus*	Rapid progression, influenza association, necrosis, pneumatoceles, or bronchopleural fistula	Vancomycin; clindamycin may be considered when local susceptibility is reliable	Susceptibility-guided therapy	Regional prevalence and inducible clindamycin resistance should inform treatment [1,11,26,27].
*Streptococcus pyogenes*	Invasive disease, bacteremia, shock, or rapidly progressive pleuropulmonary infection	Beta-lactam-based CAP regimen	Penicillin or another narrow-spectrum beta-lactam; consider toxin-suppressive therapy in severe invasive disease according to local protocols	Close monitoring is required because systemic deterioration may be rapid [11,12].
Gram-negative or anaerobic infection	Neonatal disease, aspiration, immunocompromise, trauma, postoperative infection, or healthcare-associated infection	Broaden treatment according to individual risk factors and local epidemiology	Culture- and susceptibility-directed treatment	

Abbreviation: CAP, community-acquired pneumonia.

**Table 3:** Comparative pediatric randomized trials of catheter drainage with fibrinolysis and video-assisted thoracoscopic surgery (VATS). Trial endpoints and crossover or additional interventions were not defined or reported uniformly.

Trial	Design, Endpoints, Findings, and Limitations
Sonnappa et al. [18] N = 60 (30 per arm)	**Eligibility and intervention:** Children with empyema were randomized to percutaneous chest drainage with intrapleural urokinase or primary VATS. **Endpoints and findings:** Primary endpoint: hospital days after intervention. Secondary outcomes included drain days, total hospital stay, treatment failure, 6-month radiologic outcome, and costs. Post-intervention stay was a median of 6 days in both groups; total stay and 6-month radiologic outcomes did not differ significantly. **Crossover and limitations:** Treatment failure was reported, but rescue treatment and crossover were not described using a definition directly comparable with later trials. Single-center study with a modest sample.
St Peter et al. [17] N = 36 (18 per arm)	**Eligibility and intervention:** Empyema was defined by imaging loculation or pleural-fluid leukocyte count >10,000/µL. Children received a 12-Fr tube with tPA (4 mg, three doses) or VATS. **Endpoints and findings:** Primary endpoint: hospital stay after treatment. Secondary outcomes included clinical recovery, oxygen requirement, analgesia, and charges. The primary endpoint did not differ significantly between groups. **Crossover and limitations:** Three of 18 children assigned to fibrinolysis subsequently underwent VATS for inadequate response. Small, single-center trial; the fibrinolytic regimen differed from urokinase trials.
Cobanoglu et al. [19] N = 54 (27 per arm)	**Eligibility and intervention:** Children with stage II or III empyema were randomized to VATS or chest-tube drainage with streptokinase. **Endpoints and findings:** Reported outcomes included symptom duration, chest-tube duration, hospital stay, and lung expansion. Symptom, tube, and hospital duration were shorter in the VATS group; reported lung-expansion success was 77.8% with VATS and 70.4% with fibrinolysis. **Crossover and limitations:** The abstract does not provide a directly comparable crossover definition. Single-center study with stage-based eligibility and a fibrinolytic agent different from the other trials.
Marhuenda et al. [20] N = 103 (53 VATS; 50 urokinase)	**Eligibility and intervention:** Multicenter trial of children with septated parapneumonic empyema randomized to thoracoscopy or drainage with urokinase. **Endpoints and findings:** Main outcome: hospital stay after treatment. Secondary outcomes included postoperative stay, fever after treatment, and additional intervention. Median hospital stay was 14 days with VATS and 13 days with urokinase; these and other principal recovery outcomes did not differ significantly. **Crossover and limitations:** A second intervention was required in 15% of the VATS group and 10% of the urokinase group (*p* = 0.47). Larger and multicenter, but protocols and endpoint definitions still differed from the other trials.

Interpretation. The four trials enrolled approximately 253 children in total, but differed in eligibility, fibrinolytic agent, drain protocol, endpoints, and treatment-failure or crossover reporting. Their results should therefore be interpreted as evidence of uncertainty, not as proof that the approaches are equivalent. Abbreviations: Fr, French gauge; tPA, tissue plasminogen activator; VATS, video-assisted thoracoscopic surgery.

**Table 4:** Key pediatric evidence, limitations, and practical implications. This table is an evidence summary, not a dosing guide or a substitute for local pediatric protocols.

Decision	Evidence, Limitations, and Practical Implication
Observation or drainage	**Evidence:** The 2026 IDSA/PIDS guideline suggests observation for small, uncomplicated effusions and recommends drainage for moderate effusions with respiratory distress, large effusions, or purulent fluid [6]. **Limitations:** Evidence comparing observation and drainage is mainly observational; severity and treatment selection vary across cohorts. **Practical implication:** Observe small uncomplicated effusions in clinically stable children. Base drainage decisions on clinical status, fluid characteristics, and trajectory.
Ultrasound	**Evidence:** The 2026 guideline suggests ultrasound rather than CT or MRI to characterize moderate to large effusions [14]. **Limitations:** Conditional recommendation based on very low-certainty evidence; access to skilled ultrasound varies. **Practical implication:** Use ultrasound to characterize pleural fluid and guide procedures. Obtain additional imaging when it is likely to change management.
Drain size	**Evidence:** The 2026 guideline suggests small-bore tubes (≤12 Fr) over large-bore tubes (≥14 Fr) [31]. **Limitations:** Direct comparative pediatric evidence is limited and largely observational. **Practical implication:** A small-bore catheter is appropriate for many children when correctly positioned and kept patent; follow local protocols.
Fibrinolytic agent	**Evidence:** Pediatric studies and guidelines support catheter drainage with fibrinolysis for many loculated collections [5,17,18,19,20,21,32]. **Limitations:** Regimens and escalation criteria vary; pediatric evidence has not established one agent or schedule as superior. **Practical implication:** Use an established pediatric protocol and assess clinical response and adequacy of source control.
tPA alone or tPA–DNase	**Evidence:** A pediatric randomized trial and the 2026 guideline support tPA alone over routine addition of DNase [21,22]. **Limitations:** The recommendation is conditional and relies on one pediatric randomized trial. **Practical implication:** Do not routinely add DNase to tPA for pediatric empyema.
First-line VATS	**Evidence:** The 2026 guideline suggests chest-tube drainage with fibrinolysis rather than surgical debridement as first-line treatment for most children who need drainage; VATS remains an option for selected or refractory cases [6,17,18,19,20]. **Limitations:** Comparative trials are small and heterogeneous; anatomy, severity, expertise, and resources differ. **Practical implication:** Consider VATS for selected complex cases or inadequate catheter source control; individualize the approach.
Corticosteroids	**Evidence:** Limited pediatric evidence includes a randomized dexamethasone study [29,30]. **Limitations:** Evidence is insufficient to establish routine benefit; patient selection and outcomes vary. **Practical implication:** Corticosteroids are not standard therapy; consider selectively under local clinical guidance.
Antibiotic duration	**Evidence:** The optimal duration has not been established in pediatric randomized trials; courses are individualized in current practice [1,5,26]. **Limitations:** Duration studies and criteria for IV-to-oral transition are limited and inconsistent. **Practical implication:** Individualize duration by pathogen, clinical response, complications, and source control. Do not use imaging abnormalities alone to prolong therapy.

Scope note. The 2026 IDSA/PIDS recommendations cited here apply to children aged 3 months to 18 years with community-acquired pneumonia-associated pleural effusion or empyema. Neonatal, healthcare-associated, postoperative, aspiration-related, and other non-pneumonic infections require separate age- and context-specific assessment. Abbreviations: CT, computed tomography; DNase, deoxyribonuclease; Fr, French gauge; IDSA, Infectious Diseases Society of America; IV, intravenous; MRI, magnetic resonance imaging; PIDS, Pediatric Infectious Diseases Society; tPA, tissue plasminogen activator (alteplase); VATS, video-assisted thoracoscopic surgery.

## Data Availability

No new data were created or analyzed in this study. Data sharing is not applicable to this article.
